# Sensing acidosis: nociception or sngception?

**DOI:** 10.1186/s12929-018-0486-5

**Published:** 2018-11-29

**Authors:** Jiann-Her Lin, Chih-Hsien Hung, Der-Sheng Han, Shih-Ting Chen, Cheng-Han Lee, Wei-Zen Sun, Chih-Cheng Chen

**Affiliations:** 1 Institute of Biomedical Sciences, Academia Sinica, 128 Academia Rd. Sec. 2, Taipei, 115 Taiwan; 2 Department of Neurosurgery, Taipei Medical University Hospital, Taipei, Taiwan; 3 Department of Neurology, Kaohsiung Medical University Hospital; Kaohsiung Medical University, Kaohsiung, Taiwan; 4 PhD program in Translational Medicine, Kaohsiung Medical University and Academia Sinica, Taipei, Taiwan; 5 Department of Physical Medicine and Rehabilitation, National Taiwan University Hospital, Bei-Hu Branch, Taipei, Taiwan; 6 Department of Anesthesiology, National Taiwan University Hospital, Taipei, Taiwan; 7 Taiwan Mouse Clinic – National Comprehensive Mouse Phenotyping and Drug Testing Center, Academia Sinica, Taipei, Taiwan

**Keywords:** Acidosis, ASIC3, Nociception, Pain, Sngception, Soreness

## Abstract

**Background:**

Sensing tissue acidosis is an important function of the somatosensory nervous system to response to noxious stimuli.

**Main body:**

In the pain clinic, acid or soreness sensation is a characteristic sensory phenotype of various acute and chronic pain syndromes, such as delayed onset muscle soreness, fibromyalgia, and radicular pain. However, soreness sensation is a sign of successful analgesia for acupuncture and noxipoint therapy. Thus, the nature of acid or soreness sensation is not always nociceptive (or painful) and could be anti-nociceptive. To facilitate the investigation of the molecular and neurobiological mechanisms of soreness sensation, we propose a concept called “sngception (sng- ception)” to describe the response of the somatosensory nervous system to sense tissue acidosis and to distinguish it from nociception. “Sng” is a Taiwanese word that represents the state of soreness while at the same time imitates the natural vocalization of humans feeling sore.

**Conclusion:**

Here we propose sngception as a specific somatosensory function that transmits the acid sensation from the peripheral to the central nervous system. Sngception could partially overlap with nociception, but it could also transmit antinociception, proprioception, and pruriception.

## Introduction

Tissue acidosis is a physiological or pathological phenomenon occurring during tissue injury, inflammation, ischemia, fatiguing exercise, and tumor growth [1–5]. Clinically, several types of acute and/or chronic pain are closely associated with tissue acidosis ranging from pH 7.3 to 5.8 or lower (Table 1). Thus, sensing tissue acidosis is an important function for the somatosensory nervous system to monitor and respond to the “noxious” stimulation.

**Table 1:** Types of tissue acidosis and associated pain

Conditions	Affected sites	Examples	pH ranges (in animal models)	Pain/soreness	References
Ischemia	Brain	Stroke	6.2~ 7.0 (Rat)	Central post-stroke pain	[47]
	Brain	Intracranial hypertension	~ 7.05	Headache and widespread pain	[48, 49]
	Heart	Angina	6.9~ 7.0 (Cat)	Chest pain	[4]
	Intestine	Small bowel obstruction	6.8~ 7.1	Abdominal pain	[50]
	Lower limbs	Peripheral artery disease	< 7.15 (Cat)	Pain	[51]
	Muscle	Tourniquet test	~ 7.0	Muscle pain	[9]
	Vessel	Sickle cell disease	< 7.36	Sickle pain	[52, 53]
Injury	Skin	Surgery	6.9~ 7.1 (Rat)	Postoperative pain	[5]
	Muscle	Surgery	6.5~ 7.1 (Rat)	Postoperative pain	[5]
Inflammation	Joint	Osteoarthritis	6.9~ 7.4 (Rabbit)	Pain	[54]
	Joint	Rheumatoid arthritis	7.1~ 7.4 ~ 6.2 (Mouse)	Pain	[34] [3]
	Skin	Skin wound	7.0~ 7.3 (Rabbit)	Dermal pain	[55]
	Blood	Sepsis	7.2~ 7.3	Pain/Soreness	[56]
	Muscle	Flu	NA	Pain/soreness	[57]
Fatiguing exercise	Muscle	Delayed onset muscle soreness	6.6~ 6.9 (Mouse)	Soreness	[2]
Metabolism disorders	Blood	Diabetic ketoacidosis	≤ 6.9 ~ 7.2	Abdominal pain	[58]
	Blood	Renal tubular acidosis	~ 7.2	Widespread muscle & joint pain	[59]
Tumor	Bone	Bone cancer	4.0~ 6.0 (Mouse)	Cancer pain	[60]

NA: not available

Somatosensory nerves projected from dorsal root ganglia (DRG), trigeminal ganglia (TG), nodose ganglia, etc. are responsible for detecting such noxious acidosis [6]. Many ion channels and receptors have been identified as proton-sensing receptors expressing in a variety of sensory neurons, including the pain-sensing fibers or “nociceptors”. To probe how acidosis is sensed in the somatosensory nervous system, techniques involving whole-cell patch clamp recordings of dissociated neurons are commonly used [7]. Many efforts have involved demonstrating that acidosis can effectively induce inward currents in nociceptive neurons of DRG and TG and thus depolarize the neurons to transmit the noxious acid signals to the brain where we feel the pain. Combined with the retrograde tracing technique, whole-cell patch clamp recordings have thus revealed that acid-sensitive sensory neurons innervate tissues all over the body, including skin, muscle, joint, bone, tooth, intestine, colon, and many viscera tissues [6, 8].

### Proton-sensing molecules in nociceptors

**Fig. 1:**
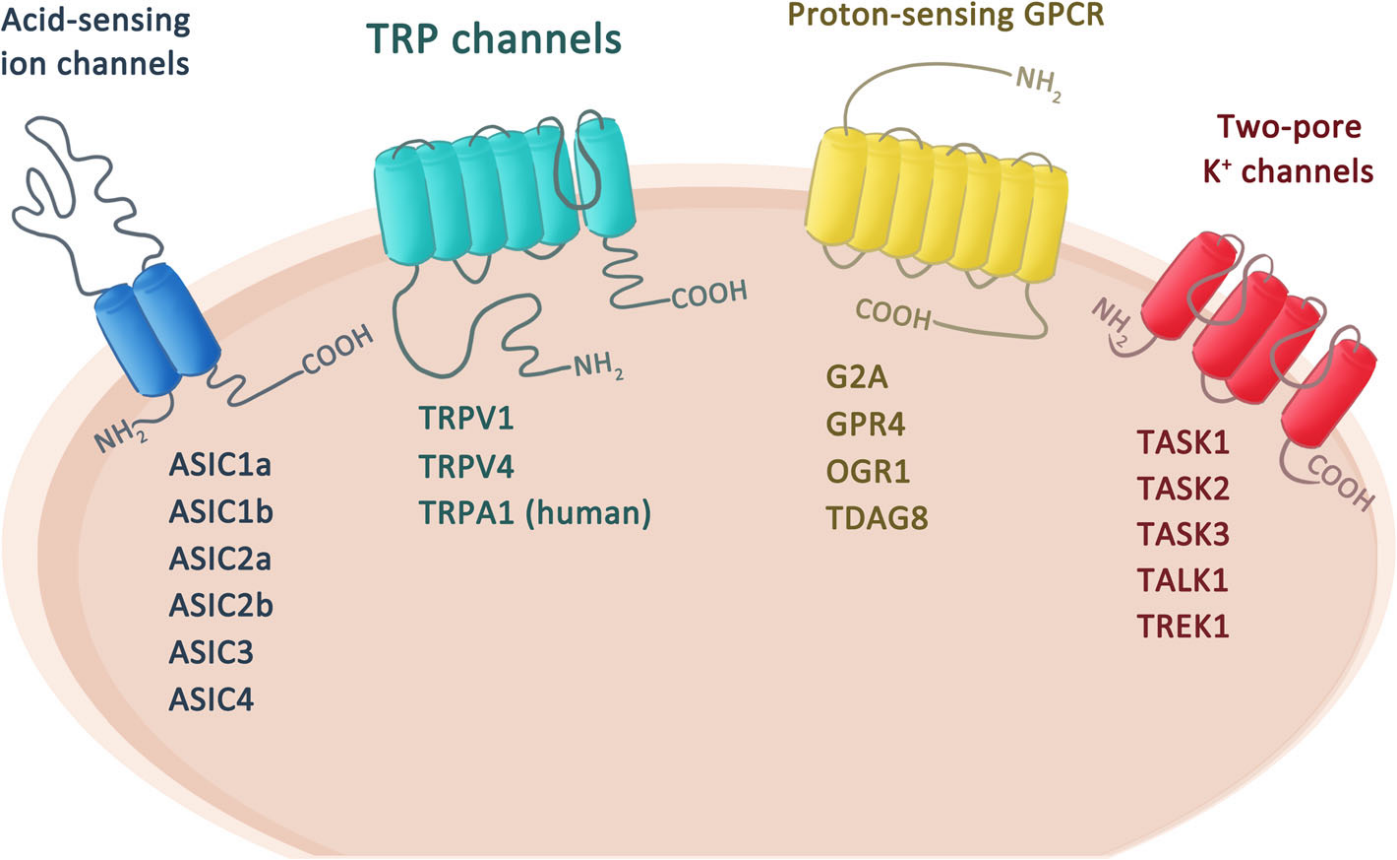
Molecular determinants that sense tissue acidosis. Proton-sensing ion channels and/or receptors expressed in nociceptors include members of acid-sensing ion channels (ASICs), transient receptor potential (TRP) channels, two-pore potassium channels (K2Ps), and protein-sensing G-protein-coupled receptors such as G2A, GRP4, OGR1, and TDAG8

Experimentally, intradermal or intramuscular acidosis is noxious and painful in humans [9–12]. However, the exact acid sensors in the nociceptors that trigger pain are still disputed. Candidate proton-sensing ion channels and/or receptors contributing to acid-induced pain include members of acid-sensing ion channels (ASICs), transient receptor potential (TRP) channels, proton-sensing G-protein-coupled receptors (e.g., GPR4, G2A, OGR1, TDAG8), and two pore potassium channels (K2Ps) (Fig. 1) [13]. Among these proton-sensing membrane proteins, ASIC3 and TRP/vanilloid receptor subtype 1 (TRPV1) are most abundantly expressed in nociceptors and thus are intensely studied for their roles in acid-induced pain in humans and animals. In rodents, pharmacological blockade or genetic deletion of ASIC3 prevents the induction of pain behaviors in models of postoperative pain, inflammatory pain, and ischemic pain [14]. In a mouse model of fibromyalgia induced by repeated intramuscular acid injections, ASIC3 and TRPV1 play essential roles in acute pain induction, hyperalgesic priming, and the development of chronic pain [15, 16].

### Does acid induce nociception or sngception?

Although accumulating evidence has shown that acid can induce nociception and trigger pain, researchers in the field of pain biology or neuroscience have oversimplified the roles of acid sensation for decades. For instance, since the earliest discovery of acid-induced currents in sensory neurons, researchers have identified the proton-activated conductance in a variety of sensory neuron subtypes including nociceptors and non-nociceptors [17]. The discovery was initiated by an original assumption: “pH is one of the most stable parameters of homeostasis — why shouldn’t there be a quick neuronal sensor capable of transforming this parameter into the receptor potential and sensory code?” [18]. Later studies found acid-induced currents in ~ 67% of total DRG neurons, which is much higher than the total number of nociceptors [7]. In particular, 85% of muscle afferent DRG neurons are acid-sensitive [15]. Obviously, many acid-sensitive DRG neurons are not nociceptors, whereas many nociceptors are not acid-sensitive [15, 19]. In knockout mouse models, lacking an ASIC subtype produces defects in acidosis-induced pain and in neurosensory mechanotransduction [6, 19]. Therefore, acid sensation cannot be included with nociception, although overlap does exist.

In clinical practice, acid sensation might be related to soreness sensation, as shown in delayed-onset muscle soreness (DOMS) [20]. Many types of pain disorders are closely related to tissue acidosis (Table 1). In addition, acidosis induced itchiness or paresthesia in many clinical studies of health volunteers [11, 21]. Moreover, experienced clinicians usually depend on inquiring about a patient’s soreness sensation as a crucial sign of whether the correct manipulation of the targeted acupoint and noxipoint is reached [22, 23]. Thus, to interpret the multiple roles of acid sensation involved in nociception, anti-nociception, and non-nociceptive sensation, we propose “sngception” to replace “nociception” to describe acid sensation and/or soreness sensation in the somatosensory system (Table 2). Briefly, “sng” is a Taiwanese word that represents the state of soreness while at the same time imitates the natural vocalization of humans feeling sore.

**Table 2:** The phenomena of pain and sng

	Pain	Sng
Trigger	Tissue damage	Tissue acidosis; mechanical stimuli
Sensation	Nociception	Sngception
Perception	Pain	Soreness (or Sng)
Responses	Suffering	Suffering; relief by passive massage and/or active stretch
Behaviors	Avoiding	Dual manifestations: (1) avoiding; (2) active relieving maneuvers (e.g., hitting, stretch, massage).

### Acid-mediated nociception and anti-nociception

Accumulating evidence has shown ASIC3 and TRPV1 as two major acid sensors in nociceptors that trigger acid-induced pain in mouse models of fibromyalgia [13, 15]. However, our recent studies have shown that acid can also mediate an anti-nociceptive effect on muscle nociceptors while both ASIC3 and TRPV1 are blocked [24]. We name this acid-mediated anti-nociception non-ASIC, non-TRPV1 acid signaling, which involves the release of substance P from muscle afferent terminals and acts on NK1R to open an M-type potassium channel (Kv7) in a G-protein-independent, tyrosine kinase-dependent manner [25]. The acid-mediated anti-nociception can tune the acid-induced nociceptor priming and thus prevent pain chronicity in fibromyalgia models [13]. Interestingly, about ~ 60% of acid-sensitive muscle afferent DRG neurons express neither ASIC3 nor TRPV1 [15]. The molecular identity of the non-ASIC3, non-TRPV1 acid sensor is not known, but possible candidates are proton-sensing receptors and/or ion channels expressed in muscle afferents, including GRP4, G2A, OGR1, TDAG8, ASIC1a, ASIC1b, ASIC2a, ASIC2b, TRPA1, TASK1~ 3, and TREK1 (Fig. 1).

**Fig. 2:**
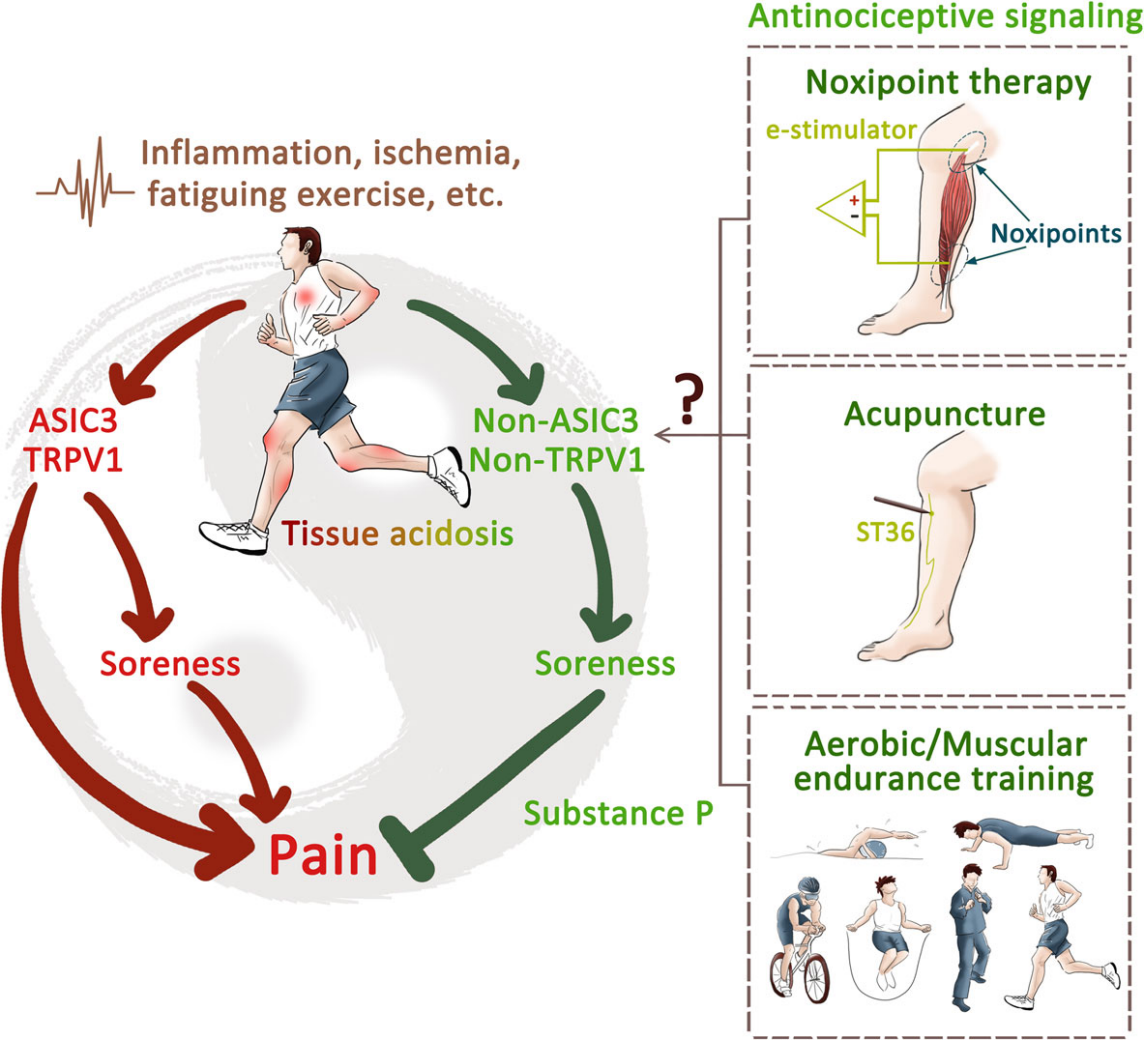
Soreness hypothesis. Tissue acidosis simultaneously evokes a pro-nociceptive signaling via ASIC3/TRPV1 activation and an anti-nociception via non-ASIC3, non-TRPV1 acid signaling. In most cases, pain associated with tissue acidosis is the balanced result of pro-nociceptive and anti-nociceptive acid signaling. In physical therapy, soreness sensation induced by acupuncture and/or noxipoint therapy is a sign of successful analgesia to balance the pro-nociceptive signaling. Fibromyalgia patients might have soreness phenotypes due to an imbalance of acid signaling in muscle nociceptors, especially with impaired non-ASIC3, non-TRPV1 acid signaling

The acid-mediated anti-nociceptive effect reminds us of a specific soreness sensation, also called De-qi (得氣), occurring in acupuncture analgesia [23]. Similarly, soreness sensation is a key factor of a novel noxipoint therapy to treat chronic neck and shoulder pain, in which analgesia can be achieved via intense electrical shock on two ends of a muscle to trigger a soreness sensation [22]. In both cases, the soreness sensation of deep tissues is a sign of anti-nociception, which may be related to the non-ASIC3, non-TRPV1 acid signaling, although it might be also nociceptive. In one-third of fibromyalgia patients who have prominent soreness phenotypes, acupuncture usually has no analgesic effect and even worsens the soreness phenotypes. Thus, here we propose that the soreness phenotype of fibromyalgia is an imbalanced acid signaling between acid-induced nociception and acid-mediated anti-nociception, mainly due to impaired non-ASIC3, non-TRPV1 acid signaling (Fig. 2). In contrast, acupuncture, noxipoint therapy, or aerobic/muscular endurance training could boost the antinociceptive acid (soreness) signaling and thus relieve pain. In sport medicine, well-trained athletes can tolerate exercise-induced muscle damage (EIMD) and muscle soreness. EIMD is scientifically and clinically accepted as an important phenomenon in sport medicine, but soreness is usually considered the primary endpoint for proper recovery from the “damage” [26]. Thus, professional coaches and athletes would develop active strengthening programs to adapt to the “soreness” and speed up the recovery process.

Although the non-ASIC3, non-TRPV1 acid sensor is a promising novel target for the development of effective analgesics, its molecular identity is not known. Thus, probing the molecular determinants that contribute to the non-ASIC3, non-TRPV1 acid signaling will be a key step to reveal the mystery of fibromyalgia, especially for individuals who have soreness phenotypes and are resistant to acupuncture analgesia.

Hence, sngception could be nociception mediated by ASIC3 and/or TRPV1 or anti-nociception mediated by non-ASIC3, non-TRPV1 acid sensors.

### Non-nociceptive acid sensation

Besides nociception and anti-nociception, acidic citric buffer (pH 3.0) induces itch responses in mouse skin and histamine-sensitized skin in humans [21]. Itch sensation is mediated by a group of somatosensory neurons named pruriceptors that express specific types of G-protein-coupled receptors (such as H1, MRGPRX1, MRGPRA3, etc.), and/or a TRP channel, (TRPV1 or TRPA1), or a mechanoreceptor responding to pruritic agents including histamine, chloroquine, serotonin, heat, mechanical stimuli, etc. [27]. Based on these findings, itch sensation can be conducted by pruriceptors based on the specificity model, by which there are peripheral sensory neurons solely activated by pruritive stimuli so that itch can be decoded in the brain following a labeled line pathway. However, since these pruriceptive neurons also express transducers that respond to noxious stimuli (e.g., TRPV1 and TRPA1), there are several models (including opponent theory, pattern theory, intensity/temporal theory, and spatial contrast theory) to reconcile contradictory findings of itch and pain studies [28–30]. The noxious acidosis-induced itch responses are mediated by TRPV1 and TDAG8 [31]. Because TDGA8 is more sensitive to acid than TRPV1, the acidosis-induced itch could fit in the spatial contrast theory, which states that itch arises from a sharp contrast between individual nociceptors firing among the surrounding silent neighbors [30]. In the context of skin acidosis, protons could be gradually diffused in the local tissue and assumed to simultaneously activate a wide range of acid-sensitive neurons, so that acid could evoke sharp contrast activation of afferents with TDAG8-TRPV1 coupling among TRPV1-positive/TDAG8-negative afferents.

Moreover, recent studies have shown ASIC3 involved in mechanotransduction of DRG proprioceptors, the low-threshold mechanoreceptors responding to force changes during muscle contraction [32]. In DRG proprioceptors, the mechanically activated currents induced by neurite stretching are largely inhibited by the ASIC3-selective antagonist APETx2 or ASIC3 gene knockout. Surprisingly, although proprioceptors are well-characterized mechanoreceptors, ASIC3 mediates an acid-induced inward current in DRG proprioceptors similar to that found in DRG nociceptors. ASIC3 was found to be a dual-function protein involved in both acid-sensing and mechano-sensing in proprioceptors, although the role of acid-sensation in proprioceptors is unknown [33].

Thus, sngception also includes TDAG8- and TRPV1-mediated itch sensation (or pruriception) and ASIC3-mediated proprioception.

### Sngception in the context of chronic pain

In clinical observations, diverse somatic complaints exist among different pain conditions. For instance, DOMS usually leads to characteristic pain-related phenotypes (e.g., hyperalgesia and allodynia) and on-going muscle soreness that results in avoidance behaviors similar to those with other types of chronic pain. Mechanistically, the activation of TRPV1-, TRPV4-, and ASIC-positive afferents is essentially involved in the induction of DOMS [20]. In inflammatory musculoskeletal pain disorders, tissue acidosis is prominent and highly associated with disease severity [34, 35]. Take rheumatoid arthritis (RA) as an example, a number of studies have shown the conditions of tissue acidosis in synovial fluid is associated with inflammatory activity and disease severity [36, 37]. Studies of RA animal models have revealed tissue acidosis inflicted by inflammation would lead to activation and up-regulation of ASIC3 on nociceptive afferents, resulting in central sensitization and pain aggravation [38, 39]. Besides, TRPV1 and TDAG8 are involved in RA-associated inflammation and RA-induced hyperalgesia in animal models [40]. Unfortunately, we know little about whether soreness is also a prominent phenotype in RA patients, although muscle soreness is reported in 24% of patients with paraneoplastic rheumatic syndromes [41]. In the majority of cases, sngception is commonly overwhelmed by co-existing nociception and thus becomes relatively less distinct. However, in our experience, soreness (or sng) is a predominant sensory phenotype in patients with fibromyalgia and radiculopathy. In our series, soreness is not only one of the major complaints in lumbar stenosis-related low back pain, but also is one of the major reasons for surgical intervention because current treatment for soreness is unsatisfactory [42]. Therefore, soreness can be another major component in chronic pain disorders but is not yet valued.

Unfortunately, we know little about sngception in the chronic pain context. Future perspective studies are warranted to assess the clinical impacts of sngception on related diseases and to evaluate their responses to therapy. Tailored clinical tools for sngception (e.g., soreness questionnaire) are needed for clinical research of these pain disorders. In addition, research into the molecular and neurobiological basis of sngception in animal models will greatly advance our understanding of the chronicity of intractable pain (and soreness) and facilitate the development of better therapeutic strategies. Because sng (soreness) is a subjective perception and can be only reported from humans, translational studies with a reverse design from the clinic to animals are imperative for phenotype elucidation and identification.

## Concluding remarks

Here we propose sngception as a specific somatosensory function that transmits the acid sensation from the peripheral to the central nervous system. Sngception could partially overlap with nociception, but it also transmits anti-nociception, proprioception, and pruriception mediated via different acid-sensitive receptors/ion channels and/or neurons with varied pH sensitivities. Similar to nociception, which can be sub-grouped into thermo-, mechano- and chemo-nociception, sngception is also composed of different sensory modalities. Further research is needed to delineate the sngception subtypes and underlying molecular mechanisms, as well as clinical impacts of sngception.

### Table 2: Nociception and sngception

In the Chinese language, “sng(acid)-pain” (痠痛), meaning a combination of soreness and pain, is much more commonly reported than “pain” among patients with chronic pain, especially for those with musculoskeletal pain. In the English dictionary, soreness means the quality of being painful and uncomfortable because of injury or too much use. However, in pain clinics, delayed-onset muscle soreness (DOMS) is probably the only type of pain that clearly highlights “soreness” occurring a few days after fatiguing exercise. DOMS results in mechanical hyperalgesia, tenderness, and movement-related muscle ache (or soreness) that lead to avoiding behaviors as with other types of chronic pain. In addition, DOMS patients show compensatory behaviors (e.g., decreased proprioception and range of motion) in response to muscle soreness, which are symptoms distinguishable from other chronic pain [43]. In other aspects, soreness sensation is commonly described in patients with chronic pain during physical therapeutic procedures, such as acupuncture and noxipoint therapy, and is a sign of De-qi (specific for acupuncture) or successful analgesia [22, 23]. Because the soreness (or acid) sensation in not always painful, it should be defined as a way to distinguish it from nociception.

*We introduce the term “sngception”* specifically to describe the response of the somatosensory nervous system to sense tissue acidosis or the activation of acid-sensitive afferent neurons. “Sng” is a Taiwanese (Southern Min) word that represents the state of soreness while at the same time imitates the natural vocalization of humans feeling sore. The word first received its Romanization form by Dutch missionaries in the 1600s and was later established by Taiwanese Language Phonetic Alphabet and the Ministry of Education of Taiwan in the early twenty-first century [44]. Starting with a voiceless alveolar fricative-“s” and a nasal velar sonorant –“ng” followed, “sng” delicately elicits the uniqueness of soreness apart from pain. In view of the importance of research into the sense of soreness, we introduce the term “sngception”, the perception of sng, for the convenience of further investigation.

#### Is there a place for soreness (sng) in pain taxonomy?

Pain is “an unpleasant sensory and emotional experience associated with actual or potential tissue damage, and described in terms of such damage” as defined by the International Association for the Study of Pain [45]. To have a better understanding of pain, Loeser described the phenomena of pain as composed of nociception, pain, suffering, and pain behaviors, with nociception being the detection of potential tissue damage by the peripheral sensory system, perception of pain resulting from a noxious input to the central nervous system, suffering a negative response caused by perceiving pain, and pain behavior anything that a person tends to do or avoid doing as a result of suffering from pain and can be measured [46]. However, there is no place for “soreness” or “sng” in the current pain taxonomy.

Is “sng” composed of different phenomena from “pain”? From our daily life experience and/or clinical observation, we propose that the phenomena of “sng” might be composed of sngception, soreness (sng), suffering (or desire for massage), and sng behaviors, with sngception being the detection of tissue acidosis (or mechanical stimulus in acupuncture) by the peripheral sensory system; sng behaviors are anything a person tends to do to compensate for suffering from “sng”. We summarize the comparison between “pain” and “sng” in Table 2.

## Abbreviations

ASIC3: Acid-sensing ion channel 3
DOMS: Delayed onset muscle soreness
DRG: Dorsal root ganglion
EIMD: Exercise-induced muscle damage
TG: Trigeminal ganglion
TRP: Transient receptor potential
TRPV1: TRP vanilloid receptor subtype 1
